# Editorial: Rethink Thoracic Surgery as a Whole After the Pandemic. How to Optimize Resources and Deliver Excellent Patient Care

**DOI:** 10.3389/fsurg.2022.920626

**Published:** 2022-05-26

**Authors:** Federico Raveglia, Riccardo Orlandi, Hecheng Li, Robert Cerfolio, John Kit Chung Tam, Marco Scarci

**Affiliations:** ^1^ Department of Thoracic Surgery, San Gerardo Hospital, Monza, Italy; ^2^ Department of Thoracic Surgery, Ruijin Hospital, School of Medicine, Shanghai Jiao Tong University, Shanghai, China; ^3^ Department of Cardiothoracic Surgery, NYU Langone Health, New York, NY, United States; ^4^ Department of Cardiac, Thoracic and Vascular Surgery, National University Heart Centre Singapore, Singapore, Singapore; ^5^ Department of Thoracic Surgery, Hammersmith Hospital, London, UK

**Keywords:** thoracic surgery, COVID-19, pandemic (COVID19), lean, six sigma (6σ), resources, optimization

## Editorial on the Research Topic 

### Rethink Thoracic Surgery as a Whole After the Pandemic. How to Optimize Resources and Deliver Excellent Patient Care

Surgical departments, especially thoracic surgery ones, are cost-intensive, multi-professional parts of health-service delivery. Managing these units efficiently is essential when hospitals and healthcare systems aim to maximize health outcomes with limited resources. In dynamic surgical environments continuous resources optimization is mandatory. Given the complexity of the many activities in a surgical department, this process necessarily involves multiple aspects ranging from technological innovation in both operating rooms and wards to the development of constructive and satisfactory collaborations between prepared and motivated staff members.

Therefore, improving thoracic surgery department efficiency can be difficult to manage, especially after a long time without change, when routines become established.

On the contrary, history teaches us that it was often the most dramatic events that provided the opportunity for many cornerstone developments, just because these events disclosed the limitations of the established system. Therefore, just as important discoveries were made in the field of medicine and surgery in wartime, so today the ongoing pandemic can be an opportunity for new progress.

The COVID-19 pandemic has had a massive impact on health services all over the world. Concerning surgical departments, the influence of COVID-19 on daily practice has been widespread, ranging from workforce and staffing issues, procedural triaging, peri and intraoperative infection risk, changes to perioperative practice, and ways of working alongside consequences on surgical education and training (1, 2).

To face the pandemic, many measures have been adopted, and if, on the one hand, many aspects of the emergency have worsened work activity, on the other hand, it has given us the opportunity to develop changes that were previously unthinkable and to rethink our departments on the basis of the experience gained.

We are aware that some dramatic emergency aspects have fortunately been closely linked to the pandemic state (especially in the pre-vaccine era) and are unlikely to recur; however the skills learned in the last 2 years will be essential to (1) manage the transition to the endemic phase of the infection and (2) introduce better resource management regardless of COVID-19.

With this goal, we have collected the experience of authoritative colleagues worldwide by addressing the following key points: (1) rational strategies to manage thoracic malignancies during the pandemic, (2) definitive innovations introduced for the future during the pandemic, (3) redefine excellence in the endemic phase of the infection, and (4) correct methodologies to define and develop strategies for innovation.

Zhang and coworkers have faced two of the most meaningful issues during the acute phase of the pandemic, especially in the pre-vaccine era, when availability for elective surgery was severely affected: the prevention of nosocomial virus transmission among patients and personnel and the scheduling of surgery for oncologic patients. In particular, based on a serious literature review, they have listed a series of measures for radiologically differential diagnosis between non solid early stage lung cancer and COVID-19. Moreover, above all, they have defined the rational criteria for surgical triage for oncologic patients both for lung and esophageal cancer. Their criteria are based on cancer stage and aggressiveness and are oriented to select the cases that most need surgery in the shortest possible time. These findings, so critical during the start of the pandemic, are currently still useful in the context of a reorganization to make the planning of surgical interventions and operating theaters more effective.

N. Pozzi and coworkers focused on new ways to deliver care in a setting of scarcity and on their contribution to a new approach to patients and colleagues from an all around point of view. In particular we appreciated two interesting issues.

The first one is the role of surgery in the management of COVID-19-related thoracic complications. Presenting their data, the authors showed that surgery with salvage intent is feasible with acceptable results in these selected populations. Their contribution is really meaningful, since even if patients with severe COVID-19 are much less numerous than in the past, it is likely that fewer numbers will still continue to be present in our intensive care departments and management of their complications will be an issue for years.

The second one is the impact of new technologies on outpatient care and surgical training during the lock-down period. In particular they focused on telemedicine and e-learning (such as simulation and virtual learning). The push given by the pandemic to the digitization of healthcare is essential with a view to optimizing resources now and in the future.

Leow and coworkers mainly focused on the challenges ahead considering that the COVID-19 pandemic is transitioning into the endemic phase. In our opinion, their paper is a good opportunity for an overview of the healthcare system to come in the near future. In particular they prompt us to consider the role of vaccination campaigns, even outside the emergency, and the advent of digitization and automation as a hot topic of the era.

Lastly, my colleagues and I have addressed the issue of how to correctly develop strategies for healthcare innovation. We introduced the role of Lean Six Sigma (LSS) in decreasing hospital stay and waiting time in lung cancer diagnosis. We also presented the role of LSS theory in cost saving while decreasing risks in thoracic surgery procedures. Our aim is to show how the interaction with a managerial scientific vision of the processes related to it is fundamental for the improvement of healthcare.

To conclude, the pandemic has been a dramatic challenge for the healthcare system in general and thoracic surgery departments as well. Even if the worst seems to be behind us, the challenges it poses to us for the near future are no less demanding (3, 4). Indeed, if only pre-pandemic capacity will be available, it will be impossible to make up for the delay. We have to change this event into an opportunity to heal first and then optimize the quality of care for our patients.

We believe that this focused research will be helpful for those colleagues who want to undertake this task which is as dutiful as it is stimulating.
